# Assessment of Clinical Diagnosis, Microscopy, Rapid Diagnostic Tests, and Polymerase Chain Reaction in the Diagnosis of *Plasmodium falciparum* in Nigeria

**DOI:** 10.1155/2013/308069

**Published:** 2013-11-24

**Authors:** Olusola Ojurongbe, Olunike Olayeni Adegbosin, Sunday Samuel Taiwo, Oyebode Armstrong Terry Alli, Olugbenga Adekunle Olowe, Taiwo Adetola Ojurongbe, Oloyede Samuel Bolaji, Oluwaseyi Adegboyega Adeyeba

**Affiliations:** ^1^Department of Medical Microbiology & Parasitology, Ladoke Akintola University of Technology, PMB 4400, Osogbo, Osun State, Nigeria; ^2^Department of Biomedical Science, Ladoke Akintola University of Technology, PMB 4400, Osogbo, Nigeria; ^3^Department of Mathematical and Physical Sciences, Osun State University, PMB 4494, Osogbo, Nigeria

## Abstract

This study compares the performance of clinical diagnosis and three laboratory diagnostic methods (thick film microscopy (TFM), rapid diagnostic test (RDT), and polymerase chain reaction (PCR)) for the diagnosis of *Plasmodium falciparum* in Nigeria. Using clinical criteria, 217 children were recruited into the study out of which 106 (48.8%) were positive by TFM, 84 (38.7%) by RDT, and 125 (57.6%) by PCR. Using a composite reference method generated from the three diagnostic methods, 71 (32.7%) patients were found to be truly infected and 90 (41.5%) truly uninfected, while 56 (25.8%) were misidentified as infected or noninfected. When each of the 3 diagnostic methods was compared with the composite reference, PCR had sensitivity of 97.3%, specificity of 62.5%, positive predictive value (PPV) of 56.8%, and negative predictive value (NPV) of 97.8%; microscopy had sensitivity of 77.2%, specificity of 72%, PPV of 66.9%, and NPV of 81.1%, while RDT had sensitivity of 62.3%, specificity of 87.4%, PPV of 67.7%, and NPV of 84.5%. PCR test performed best among the three methods followed by TFM and RDT in that order. The result of this study shows that clinical diagnosis cannot be relied upon for accurate diagnosis of *P. falciparum* in endemic areas.

## 1. Introduction

Malaria remains an important public health concern in countries where transmission occurs regularly as well as in areas where transmission has been largely controlled or eliminated. It was estimated that there are 39 million children under 5 years of age who experience 33.7 million malaria episodes and 152,000 childhood deaths from malaria each year in areas suitable for seasonal malaria chemoprevention [1]. Factors such as drug pressure, strain variation, or approaches to blood collection affect the morphological appearance of malaria species which have created diagnostic problems that invariably had a negative effect on malaria control [2]. With the introduction of high cost antimalarial (artemisinin based therapies) the need for accurate diagnostic tools for monitoring malaria elimination/eradication successes becomes a task that must be achieved [3, 4]. 

In most endemic countries malaria diagnosis depends mainly on clinical evidence and in some cases thick film microscopy (TFM) and rapid diagnostic technique (RDT) may be used for laboratory confirmation. Microscopy remains the gold standard for malaria diagnosis and it is less costly with a threshold sensitivity of 5 to 50 parasite/*μ*L (depending on the microscopist expertise) [5]. Microscopy can also characterize the infecting species and also determine their relative densities [6]. The major constraints of microscopy include the requirement of considerable technical expertise and the fact that it is time-consuming for optimal blood film preparation, examination and interpretation [6]. RDT, an immunochromatographic capture procedure was developed to improve the timeless sensitivity, and objectivity of malaria diagnosis through less reliance on expert microscopy [2]. Preferred targeted antigens for RDTs are those which are abundant in all asexual and sexual stages of the parasite. Currently the focus of RDT is on the detection of Histidine-Rich Protein2 (HRP-2) from *Plasmodium falciparum *and Parasite-Specific Lactate Dehydrogenase (pLDH) or *Plasmodium* aldolase from the parasite glycolytic pathway found in all species [7]. However, several factors in the manufacturing process as well as environmental conditions may affect RDT performance, and these include suboptimal sensitivity at low parasite densities, inability to accurately identify parasites to the species level or quantify infection density, and a higher unit cost relative to microscopy [8]. Polymerase chain reaction (PCR), another diagnostic technique, detects specific nucleic-acid sequence and its values lie in its sensitivity, with the ability to detect five parasites or less/*μ*L of blood [9]. PCR is useful both for initial parasite diagnosis and for followup during drug efficacy study [10]. It is also useful as a sensitive standard against which other non-molecular methods can been evaluated [11]. However it is expensive and time-consuming and because of the amount of resources needed in the running of the PCR laboratory, it is used more for research purposes.

Clinical diagnosis is imprecise but remains the basis for therapeutic care for the majority of febrile patients in malaria endemic areas, where laboratory support is often out of reach. Clinical diagnosis also referred to as presumptive diagnosis is the least expensive and most commonly used method and is the basis for self-treatment in endemic countries. Overlap of malaria symptoms with other tropical diseases like typhoid fever, respiratory tract infections and viral infections impairs the specificity of presumptive diagnosis thereby encouraging indiscriminate use of antimalarials in endemic areas. Accuracy of clinical diagnosis varies with the level of endemicity, malaria season, and age group. Therefore no single clinical algorithm can be regarded as a universal predictor [12].

This paper reports the comparative performance of clinical diagnosis, TFM, RDT, and PCR in the diagnosis of *P. falciparum* malaria in Nigeria. 

## 2. Methods

### 2.1. Study Areas and Patients

The study was carried out in Osogbo located in the Western part of Nigeria. Osogbo is the state capital of Osun State, Nigeria, and it represents a typical urban setting in Nigeria. Malaria is present throughout the year with a marked increase during the raining season. Patients (ages 4 months to 20 years) who were clinically diagnosed for malaria at the outpatient departments of General Hospital Asubiaro and LAUTECH Health Centre in Osogbo were recruited into the study. Exclusion criteria used were complete absence of malaria symptoms and unwillingness to participate. All the patients that were clinically diagnosed were subsequently confirmed using TFM, RDT, and PCR before treatment. Ethical approval was obtained from the ethical committee of Osun State Hospital Management Board, Osogbo.

### 2.2. Clinical Diagnosis

Clinical diagnosis based on fever (temperature ≥ 37.5°C) and/or history of fever was carried out by physicians at the outpatient departments of the hospitals. Other symptoms considered for clinical diagnosis include headache, joint pains, body weakness, cough, diarrhea, loss of appetite/refusal of feeds, abdominal pain, and generalized body weakness.

### 2.3. Blood Collection and Analysis of RDT and Microscopy

5 mL of blood was collected aseptically from antecubital vein of consenting febrile patients, into EDTA bottle. RDT was performed on about 5 *μ*L of blood using Paracheck (Orchid Biomedical System, Verna, Goa, India) according to manufacturer's instruction. A drop of blood was used for microscopic examination of malaria parasites using thick films method stained with 5% Giemsa for 30 minutes. Parasites were counted against 200 white blood cells (WBCs) from the thick film. The parasite density was obtained by assuming a total WBC count of 8000/mL and 4.5 million RBC/mL and at least 200 fields were examined before being taken as a negative result [13]. 

### 2.4. Stevor PCR Method

10 *μ*L of blood was dotted on Whatman 3 mm filter paper and air-dried at room temperature for PCR. Parasite genomic DNA was extracted from blood samples collected on filter paper using methanol extraction method as previously described [14]. PCR was carried out using primer pairs that target the multicopy *P. falciparum* stevor gene. The PCR reaction involves a primary and nested reaction to enhance specificity. Primary amplification was performed with reaction mixture of 25 *μ*L containing 2.5 *μ*L 10x reaction buffer, 5 *μ*L of Magnesium chloride, 0.75 *μ*L of each primers (P5, P18, P20, P19), 0.2 *μ*L of DNTPs, 9.05 *μ*L of water, 0.25 *μ*L of Taq polymerase, and 5 *μ*L of DNA extract. The PCR programme was as follows: 93°C for 3 minutes, 22 cycles of 30 seconds at 93°C, 50 sec at 50°C, and 30 sec at 72° and final extension period of 3 minutes at 72°C. 2.0 *μ*L of the first PCR product was used in the second round amplification which was performed with a reaction mixture of 25 *μ*L containing 2.5 *μ*L 10x reaction buffer, 2.5 *μ*L of magnesium chloride, 0.4 *μ*L of each DNTPs, 0.25 *μ*L of Taq polymerase, 1.0 *μ*L of each primers (P24, P17), and 15.35 *μ*L of water. DNA extracted from FCR *P. falciparum* laboratory adapted strain was used as positive control and water as negative control. PCR products were subjected to electrophoresis on 1.5% agarose gels and visualized using Syngene gel documentation system (Syngene, Cambridge, UK) after staining with ethidium bromide. The primer sequences for the stevor PCR are as previously described [15].

### 2.5. Data Analysis

Data obtained was analyzed using SPSS package version 16.0. The sensitivity, specificity, and predictive values of each of the three test methods were calculated by comparing to a composite reference gold standard generated from the three methods. The composite reference method was defined as a method that is positive for malaria parasites by all the three methods (TFM, RDT, and PCR) and also negative for malaria parasites by all the three methods. This gives the method 100% hypothetical sensitivity, specificity, and positive and negative predictive values. The sensitivity, specificity, and predictive values of each of the 3 methods were then calculated using the formulas

(1) $$\begin{array}{c} \text{Sensitivity}=\frac{\text{TP}}{\left(\text{TP}+\text{FN}\right)}\times 100\\ \text{Specificity}=\frac{\text{TN}}{\left(\text{TN}+\text{FP}\right)}\times 100\\ \text{PPV}=\frac{\text{TP}}{\left(\text{TP}+\text{FP}\right)}\times 100\\ \text{NPV}=\frac{\text{TN}}{\left(\text{TN}+\text{FN}\right)}\times 100, \end{array}$$

where TP = true positive, FP = false positive, TN = true negative, and FN = false negative. Sensitivity was defined as the probability that a truly infected individual will test positive and specificity as the probability that a truly uninfected individual will test negative.

## 3. Results

### 3.1. Prevalence of Malaria Infections, Measured by the Three Diagnostic Methods

We compared the diagnostic value of 3 methods (TFM, RDT, and PCR) for the detection of malaria parasites in Nigeria. A total of 217 individuals clinically diagnosed for malaria were recruited into the study. Of these, 103 were males and 114 were with a male to female ratio of 0.9. The mean age of the patients was 8 years ± 3.04 and the mean axillary temperature was 38.2°C ± 0.96. The general characteristics of the patients are shown in Table 1. One hundred and six (48.8%) individuals were positive for malaria by TFM, 84 (38.7%) by RDT, and 125 (57.6%) by PCR. There were significant differences (*P* = 0.0005) when the prevalence of 3 methods (TFM, RDT, and PCR) was compared (Table 1). 

Using a composite reference (gold standard) method generated from the three diagnostic methods, only 71 (32.7%) patients were found to be truly infected, with *P. falciparum* 90 (41.5%) truly uninfected while 56 (25.8%) were misidentified as infected or noninfected by the three methods. When each of the 3 diagnostic methods was compared with the composite reference method, PCR had sensitivity of 97.3%, specificity of 62.5%, positive predictive value (PPV) of 56.8%, and negative predictive value (NPV) of 97.8%; microscopy had sensitivity of 77.2%, specificity of 72%, PPV of 66.9%, and NPV of 81.1%, while RDT had sensitivity of 62.3%, specificity of 87.4%, PPV of 67.7%, and NPV of 84.5% (Table 2).

Correlation of RDT and PCR to parasite density observed by microscopy is shown in Table 3. Out of 109 patients that were negative by microscopy 22 and 29 were positive for RDT and PCR, respectively. The parasite count range 101–1000 had the highest positivity (81) for microscopy. Out of this 81 microscopy positive patients, 47 and 73 patients were detected by RDT and PCR, respectively (Table 3).

## 4. Discussion

This study provides a dataset for judging the performance of clinical diagnosis against TFM, RDT, and PCR for the detection of* P. falciparum* in a malaria endemic area. Each of these methods has particular attributes that stands them out in different settings. Clinical diagnosis, for instance, is commonly used because it is cheap and allows for prompt treatment of the patient [16]. Nonspecific symptoms like fever, headache, weakness, myalgia, chills, dizziness, abdominal pain, diarrhea, nausea, vomiting, anorexia, and pruritus and other malaria related symptoms are used as the basis for clinical diagnosis. Microscopy remains the gold standard for malaria diagnosis; it is less expensive compared to other laboratory methods but has a low sensitivity. It requires well trained microscopist and when this is not present the result will not be reproducible, there will be variable sensitivity and unacceptably high false-positive rates [12]. RDTs are antigen capture tests that have been shown to be capable of detecting >100 parasites/*μ*L (0.002% parasitemia) and of giving rapid results (15 to 20 min) [8]. They are commercially available in kit form and the ease of performance of the procedures does not require extensive training, equipment, or difficulty in result interpretation [17]. The main drawback is in its specificity as parasite antigen could persist in the blood of the patient after parasite clearance by chemotherapy thereby producing false positive. PCR values lie in its high sensitivity, with the ability to detect five parasites or less/*μ*L of blood [15, 17]; however it is expensive and time-consuming.

Our results show that the continuous practice of using clinical diagnosis as the basis for antimalarial treatment in endemic area is by far not an effective diagnostic method in our study area. Out of the 217 (100%) patients that were clinically diagnosed for malaria, 104 (49.8%), 83 (38.2%), and 123 (56.7%) were positive by TFM, RDT, and PCR, respectively. Invariably irrespective of the laboratory method, about half of the patients who were diagnosed as having malaria through clinical diagnosis (syndrome approach) and who should have received antimalarial turned out to be parasite-negative. There is therefore an urgent need to review the clinical diagnosis procedure. Although it may be argued that in some cases especially in children, promptness of malaria treatments reduces the progression of simple malaria to severe malaria, which still encourages syndromic approach to malaria diagnosis. Nevertheless malaria over diagnosis is still a major public health problem in Africa with studies suggesting between 50% and 99% of those prescribed antimalarial to be test negatives depending on endemicity of the clinical setting [5, 18, 19]. The ability to rule out malaria can help to better diagnose and treat other causes of fever such as acute respiratory infection, typhoid fever, and meningitis and also avoid exposing those without malaria to drug and restricting antimalarial use to true test-positives. Till date, many clinicians in this study area still depend largely on clinical diagnosis. Our study confirmed that continual dependence on this method will lead to overdiagnosis of malaria which will result into drug wastage and encourage antimalarial drug resistance. 

In this study routine microscopic examination of Giemsa-stained blood smears which is considered as the gold standard for malaria diagnosis had a sensitivity of 77.2% and was able to detect more parasites than the RDT (sensitivity 62.3%). Though the specificity of microscopy (72%) was not as high as that of RDT (87.4%); nevertheless, it has high sensitivity, possibility for quantification of parasitemia, and easy handling which is a good advantage. Detection of parasites depends on many factors including the amount of blood processed and the competence of the microscopist, among others [20]. Also the information obtained by microscopy is limited when parasite levels are very low or when parasite morphology is altered [8]. The development of rapid diagnostic assays has attempted to address some of these shortcomings of microscopy. However it has low sensitivity at parasitemia below 100 parasites/*μ*L and have insufficient accuracy [21]. RDTs have the potential to improve the accuracy and time needed for malaria diagnosis particularly for laboratories in low or nonendemic countries, where expertise with microscopy may be limited. Major advantages of RDTs include the fact that it can be performed close to home in settings with no sophisticated infrastructure, and they do not require much skill although some level of training is needed in order for RDTs to be used properly.

Different PCR based methods have been constantly shown to be powerful tools for malaria diagnosis with better sensitivity than conventional microscopy and antigen-based diagnostic tests [18, 22]. Most positive cases were detected by the stevor PCR in this study and this method has been reported to be at least 100-fold more sensitive than other PCR assays [15, 23]. Generally, PCR has proven to be a sensitive method for diagnosis of all four species of human malaria parasites. The detection of <5 parasite/*μ*L and identification to the species level make this an excellent technique against which to compare the sensitivity and specificity of other nonmolecular methods [24].

Greater percentage of children presented at general outpatient department of the hospital in our study with fever were diagnosed for malaria (PCR—56.7%, microscopy 49.8%, and RDT 38.2%). Available records also show that at least 50% of the population of Nigeria suffer from at least one episode of malaria each year accounting for over 45% of all out-patient visits [25]. The implication of this is that malaria is still a public health problem in this area. More concerted effort is needed by government and all stake holders involved in malaria control if the goal of eradicating malaria by 2015 is to be achieved.

In conclusion our study revealed the need for complete shift from symptom-based diagnosis to parasite-based diagnosis. This can bring significant improvement to tropical fever management and reduce drug wastage and also help to curtail development of malaria drug resistance.

## Figures and Tables

**Table 1:** Characteristics of study subjects and prevalence of malaria based on different diagnostic methods.

Number of subjects	217
Mean age (years)	8 years ± 3.04
Sex male/female	103/114
Mean temperature °C	38.2°C (±0.96)
No. positive by microscopy (%), MPD ± SD	106 (48.8%), 1579.21 ± 7869.29
No. positive by RDT (%)	84 (38.7%)

No. positive by stevor PCR (%)	125 (57.6%)

MPD: mean parasite density by microscopy.

Microscopy versus RDT versus PCR = *P* = 0.0005.

**Table 2:** Sensitivity, specificity, and predictive values of the three diagnostic methods.

Diagnostic methods	Parameter for assessment							
	TP (no)	FP (no)	TN (no)	FN (no)	Sensitivity (%)	Specificity (%)	PPV (%)	NPV (%)
TFM	71	35	90	21	77.2	72	67	81.1
RDT	71	13	90	43	62.3	87.4	84.5	67.7
PCR	71	54	90	2	97.3	62.5	56.8	97.8
Composite reference	71	0	90	0	100	100	100	100

TP: true positive; FP: false positive; TN: true negative; FN: false negative; TFM: thick film microscopy;

RDT: rapid diagnostic test; PCR: polymerase chain reaction; no: number; %: percent.

**Table 3:** Stratification by parasite density in thick blood smear and correlation with rapid diagnostic test (RDT) and stevor PCR.

	Parasite count range			
	0	1–100	101–1000	**>1000**
No. observed	109	10	81	**17**
Mean parasite count/*µ*L (range)	0	91 (41.6–100)	408 (110–948)	**8,101 (1,050–81,600)**
No. positive for clinical	109 (100%)	10 (100%)	81 (100%)	**17 (100%)**
No. negative for clinical	0	0	0	**0**
No. positive for RDT	22 (20.2%)	2 (20.0%)	47 (58.0%)	**12 (70.6%)**
No. negative for RDT	87 (79.8%)	8 (80.0%)	34 (42.0%)	**4 (23.5%)**
No. positive for PCR	29 (26.6%)	8 (80.0%)	73 (90.1%)	**13 (76.5)**

No. negative for PCR	**80 (73.4%)**	**2 (20.0)**	**8 (9.9%)**	**4 (25.5%)**
